# Parenteral Nutrition in Patients with Inflammatory Bowel Disease Systematic Review, Meta-Analysis and Meta-Regression

**DOI:** 10.3390/nu11122865

**Published:** 2019-11-22

**Authors:** Jose M. Comeche, Iris Comino, Cesare Altavilla, Jose Tuells, Ana Gutierrez-Hervas, Pablo Caballero

**Affiliations:** 1 Department of Community Nursing, Preventive Medicine and Public Health and History of Science, University of Alicante, San Vicente del Raspeig, 03690 Alicante, Spain; josemiguelcomeche@gmail.com (J.M.C.); iscomino@gmail.com (I.C.); eatingfaster@gmail.com (C.A.); tuells@ua.es (J.T.); pablo.caballero@ua.es (P.C.); 2 Department of Nursing, University of Alicante, San Vicente del Raspeig, 03690 Alicante, Spain

**Keywords:** inflammatory bowel diseases, parenteral nutrition, systematic review, meta-analysis, Crohn disease

## Abstract

Inflammatory bowel disease (IBD) is a chronic disease mediated by the immune system and characterized by the inflammation of the gastrointestinal tract. This study is to understand how the use of parenteral nutrition (PN) can affect the adult population diagnosed with IBD. We conducted a systematic review, meta-analysis, and meta-regression. From the different databases (MEDLINE, Scopus, Cochrane, LILACS, CINAHL, WOS), we found 119 registers with an accuracy of 16% (19 registers). After a full-text review, only 15 research studies were selected for qualitative synthesis and 10 for meta-analysis and meta-regression. The variables used were Crohn’s Disease Activity Index (CDAI), albumin, body weight (BW), and postoperative complications (COM). PN has shown to have efficacy for the treatment of IBD and is compatible with other medicines. The CDAI and albumin improve, although the effect of PN is greater after a while. However, the effect on the albumin could be less than the observed value in the meta-analysis due to possible publication bias. The BW does not change after intervention. COM utilizing PN has been observed, although the proportion is low. More studies specifically referring to ulcerative colitis (UC) and Crohn’s disease (CD) are needed to develop more concrete clinical results.

## 1. Introduction

During the last decades, the prevalence of inflammatory bowel disease (IBD) has increased in the U.S. and Europe [1]. Moreover, it has also increased in developing countries [2,3]; thus, IBD can be considered a common disease in wide areas of the world. 

IBD is a chronic inflammatory disease mediated by the immune system. IBD includes Crohn’s disease (CD) and ulcerative colitis (UC). The altered response of the immune system leads to the inflammation of the gastrointestinal tract clinically defined by relapsing and remitting episodes [4,5]. The inflammatory process is characterized by a long-term overproduction of pro-inflammatory factors and an enhanced intestinal permeability [6]. IBD involves an inflammatory process of the intestinal layers and could cause abdominal swelling, fever, fatigue, weight loss, abdominal pain, diarrhea, bloody feces, etc. [7].

The European Society for Clinical Nutrition and Metabolism (ESPEN) has presented the guidelines and recommendations on clinical nutrition for the IBD [8]. The etiology of the IBD is not still completely understood [9,10]. Many studies have indicated that the genetic predisposition, diet, the environment, the intestinal microbial flora, and the immune responses are involved in the pathogenesis of IBD [4,5,11,12].

Diet and intestinal microbial flora could change the inflammatory response of the gastrointestinal tract [13,14]. Diet may reduce the symptoms and prevent the degenerative process of the IBD [15]. Therefore, it is considered a therapy for IBD [13]. Among the dietetic therapies for IBD, intestinal rest by parenteral nutrition (PN) is considered a strategy to reduce the inflammatory response of intestinal layers [16] and to recover from nutritional impairment [17]. The American Society for Parenteral and Enteral Nutrition (ASPEN) and ESPEN have described the use of PN [18,19,20]. PN could be considered a third way for human nutrition after oral intake and enteral nutrition. However, a combination of them has been studied [21]. PN could not advantage in IBD compared to other nutrition therapies. However, when the IBD patients are temporarily unable to receive significant oral or enteral nutrients, PN could be used as a nutritional treatment [21,22]. Also, in severe cases of IBD with surgical resection or bowel severe complications, PN could provide a supply of nutrients to maintain good nutritional status and reduce inflammatory reactions [23].

The aim of this review was to understand the use of PN and its effects on adults diagnosed with IBD.

## 2. Materials and Methods 

To achieve this objective, a systematic review was conducted in agreement with the procedures and verification list described by PRISMA [24]. Afterward, a meta-analysis on the more common results, and a meta-regression with the co-variables, surgery (Yes/no), observed moment (days), and period of treatment (days), were conducted.

### 2.1. Systematic Review

A search of scientific works was conducted in the MEDLINE database, through the system of open retrieval system on the Internet such as PubMed, Cochrane, Scopus, Web of Science, CINAHL, and LILACS. The studies conducted over time, up to 8 July 2019, were compiled.

#### 2.1.1. Inclusion and Exclusion Criteria

The studies selected had to comply with the following inclusion criteria: refer to an adult population (older than 18) diagnosed with IBD; study the effect of PN within IBD; be clinical trials; in English, Spanish, Portuguese, French, or German languages.

The following articles were excluded: those that referred to the infant population; to animals, to the use of PN in a healthy adult population; those that sought the effect of oral exclusion diets on IBD; that were observational studies; that were based on secondary sources.

#### 2.1.2. Search Equation

To include content linked to the intervention, PN, a specific descriptor was used (MeSH), such as “Parenteral Nutrition, Total”, and the term “Parenteral Nutrition” in the title or abstract.

For the content linked to the population, we utilized the descriptor that referred to the disease “Inflammatory bowel diseases”, and its equivalent term in the title or abstract.

Also, the filters “Humans”, “Adult”, and “Clinical Trial” were utilized to achieve our objective. 

Therefore, the main search equation designed for this study was:

((“Inflammatory Bowel Diseases” [Mesh] OR “Inflammatory Bowel Diseases” [Title/Abstract]) AND (“Parenteral Nutrition, Total” [Mesh] OR “Parenteral Nutrition” [Title/Abstract])) AND (Clinical Trial [ptyp] AND Humans [Mesh] AND adult [MeSH]) 

The search equation was adapted to each, and all of the databases described previously. The process was conducted between the months of June and July 2019.

#### 2.1.3. Selection Process

After eliminating duplicate records, the process of selection was conducted in two phases. The first consisted of reviewing the titles and abstracts of all the article records resulting from the adapted search equations and shown by the databases by using the inclusion and exclusion criteria and the objective of the study as the screening measure. The screening and selection of the records/articles were conducted independently by the two researchers, both experts in the fields of nutrition. These researchers agreed on the discrepancies found in order to define the final suitability of the records/articles found in the databases. The precision of the search was calculated based on the ratio of the full-text articles selected for the review divided by the number of records found by the search equation, multiplied by one hundred.

The second phase was conducted by applying the inclusion/exclusion criteria to the complete text of all the scientific studies selected in the first phase, thus ensuring the relevance of each one of them.

#### 2.1.4. Evaluation of the Quality of the Studies

The evaluation of the methodological quality of the included studies was performed by two independent researchers, using the CONSORT (Consolidated Standards of Reporting Trials) guide for clinical trials. This guide contains a list of 25 essential aspects that should be described in the publication of these studies. For each selected study, one point was assigned for each item present (if not applicable, it did not score). When an item was composed of several points, these were evaluated independently, giving the same value to each of them and subsequently an average was made (being the final result of that item), so that in no case could it beat the score of one point per item [25,26].

### 2.2. Meta-Analysis and Meta-Regression 

To calculate the effect size of the enteral nutrition on the variables, Crohn’s Disease Activity Index (CDAI), albumin, postoperative complications (COM), and body weight (BW), a meta-analysis was performed. For this, the model of fixed effects and the model of random effects were utilized. The results were presented as a forest-plot, along with the percent Heterogeneity and its confidence interval at 95%, the T-value, and the heterogeneity test.

To explore the influence of each study over the effect size, we used a leave-one-out method; pooled estimates were calculated omitting one study at a time. In addition, we plotted a scatter plot introduced by Baujat et al. [27]. On the x-axis, the contribution of each study to the overall heterogeneity statistic is plotted. On the y-axis, the standardized difference of the overall treatment effect with and without each study is plotted; this quantity describes the influence of each study on the overall treatment effect. Therefore, studies that fall in the top right quadrant of the Baujat plot have the most influence. 

Publication bias occurs when only favorable results are published, and this could have consequences on the results of the meta-analyses if these were included. To analyze the publication bias, a non-parametric analysis was conducted as proposed by Duval and Tweedie [28] based on the funnel-plot, estimating, and adjusting for the number and outcomes of missing studies in the meta-analysis. Another less-conservative proposal to estimate the number and outcomes of missing studies is the proposal by Copas et al. [29]. 

The meta-regression was utilized to understand if the duration of the intervention (days) or the surgery (yes/no) or observed moment (days) modified the effect size of the resulting variables CDAI, albumin, and BW. The effect size of COM was only related to the duration of the intervention. All the calculations were conducted within an R programming environment utilizing the packages meta version 4.9-6 [30] and metasens version 0.4-0 [31].

## 3. Results

### 3.1. Systematic Review 

**Figure 1:**
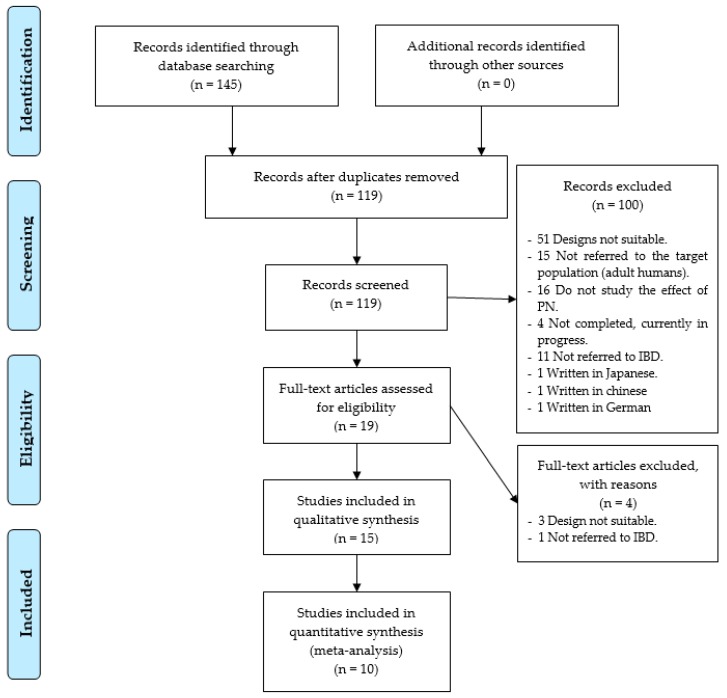
Identification and selection of studies/records in the databases.

As a result of the specific search equations used on the different databases, a total of 145 records were found of scientific articles. A total of 26 records were duplicated, leaving a total of 119 records without duplication. In the first phase of the study, exactly 100 study records were discarded, leaving 19 full-text studies to review, so that the accuracy was 16%. The reasons for not including them were that 51 records showed that the study utilized a design that was not adequate, 15 did not use an adult population, 16 did not study the effect of PN, three were written in another language other than the ones cited above, (one in Japanese, one in Chinese, and one in German), 11 did not refer to the IBD, and four were still being conducted without showing results (Figure 1).

In the second phase, four trials were removed, three due to defects in its design, and one, because the patients studied, were not diagnosed with IBD. Therefore, only 15 research studies [32,33,34,35,36,37,38,39,40,41,42,43,44,45,46] were selected, as shown in Figure 1.

**Figure 2:**
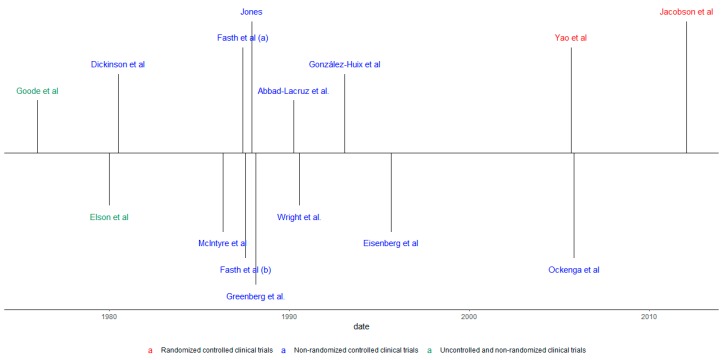
Chronological review according to the type of study and population.

As for the designs of the studies included, 11were controlled and randomized clinical studies (73.4%), two non-randomized, controlled clinical trials (13.3%), and two non-randomized, non-controlled clinical trials (13.3%) were found. In addition, six of the studies found showed results that specifically referred to CD, one study to UC, and 11 studies had results on UC and CD, under the category of IBD. Also, 10 studies mentioned the results of the disease in its active form and five studies report disease outcomes in patients under surgery. Figure 2 shows this information in a chronological manner.

As for the variety of the components of formulas employed, each study used its own formulas, normally supplemented with vitamins, minerals, and electrolytes. However, some studies employed the following commercial components/complements: “Freamine^®^”, “Amigen^®^”, “Uniasa^®^”, “Dipeptamin^®^”, “Aminoplasmal^®^”, “Vamin^®^”, “Addamel^®^”, “Soluvit^®^”, and “Vitalipid^®^”. 

In addition, a total of four types of objectives were found: six studies sought to compare the administration of PN with other techniques such as dextrose and electrolyte solutions, intravenous transfusions or oral diet, as long as possible; five studies compared PN with EN, among which, three with elemental formulas, and two with polymeric formulas; two studies sought to experiment with PN and two studies compared the same PN, but with some different component/form of withdrawal.

As for the manner of administration of the PN, the research studies generally employed a central venous catheter with the aid of an infusion pump.

The total population analyzed in the research studies found included a total of 557 individuals with IBD, with 382 diagnosed with CD, and 152 with UC.

The main tools utilized by the researchers to obtain the results were the scores, biomarkers, and tests to measure the activity of the disease—the CDAI, the Van Hees activity index (VHAI), the Truelove and Witts index; biomarkers such as CRP, ESR, the white blood cell count (WBC), levels of albumin, pre-albumin, transferrin, hemoglobin, platelet count, total bilirubin, alkaline phosphatase, etc.; and medical tests, such as the ileocolonoscopy. Complementary tests, such as urine and feces samples. Tests to measure the body’s composition, such as anthropometries and bioimpedance, to obtain parameters such as body weight (BW), triceps skinfold thickness (TSF), mid-arm muscle circumference (MAMC), etc. The follow-up of complications by health professionals, whether postoperative or during experimentation, such as infections, septicemias, cases of pneumonia, intestinal obstructions, pancreatitis, fever, hypoglycemia, hyperglycemia, etc.

Table 1 shows the main results schematically, found in the selected articles and Table 2 shows the scores obtained by the studies for their methodological quality according to the CONSORT guide. 

### 3.2. Meta-Analysis and Meta-Regression

**Figure 3:**
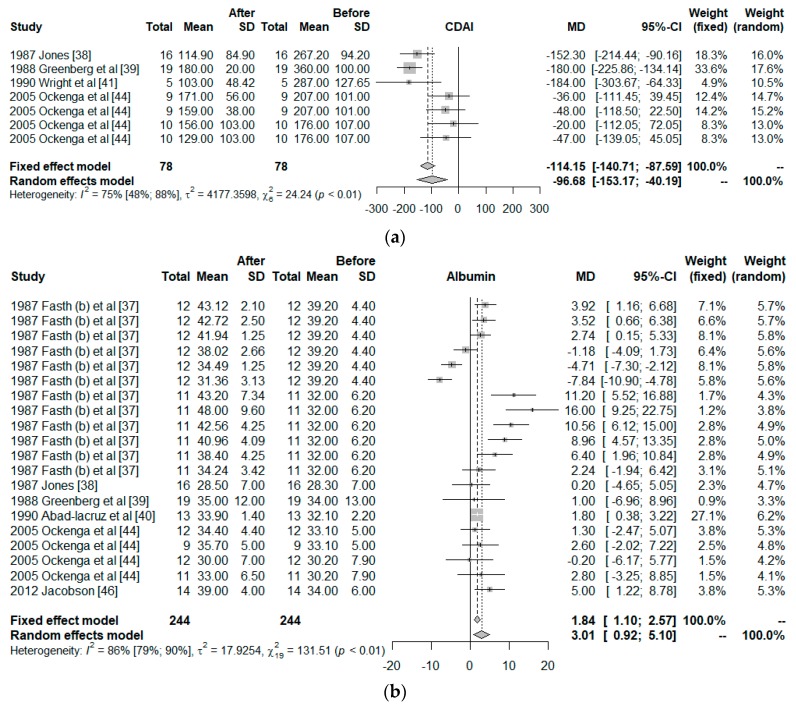

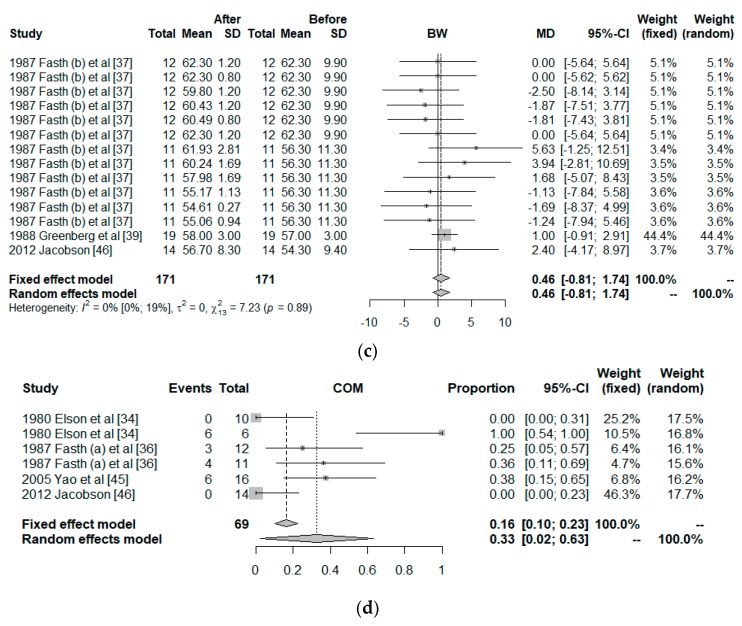
Forest plot for: (**a**) Crohn’s Disease Activity Index (CDAI), (**b**) Albumin, (**c**) Body weight (BW), and (**d**) Postoperative complications (COM).

Only 10 clinical trials had common quality and variables needed to be used in the meta-analysis. These 10 trials worked with a total of 26 groups. The final size of the sample was comprised by 298 observed moments for 164 individuals, all with IBD, to which a PN treatment had been given. The common variables were the CDAI, albumin, BW, and COM, and the co-variables; duration of the intervention, surgery, and observed moment. Figure 3 shows the effect size of the use of PN. For the CDAI and albumin, the effects are positive when comparing the situation at the start and finish of the treatment with PN, independently of whether the situation with fixed effects (less probable) or random effects (more acceptable) was considered. However, for BW, the use of PN was not significant. For COM, the effect size was significantly different from zero but the 95% confidence interval was close to zero ([0.02; 0.63]). 

The influence of each study on the results of the meta-analysis are shown in Table 3, considering a model of random effects. For CDAI, the study of Greenberg et al. has a strong effect on the results, increasing the effect size of the PN on the CDAI. The outcomes of Fasth et al. study has the strongest influence on the albumin, but it is only 5%. All studies are homogeneous for the BW. Elson et al. worked with two groups, one showed 0/10 of COM but the other showed 6/6; therefore, their study has a strong influence on the results. 

**Figure 4:**
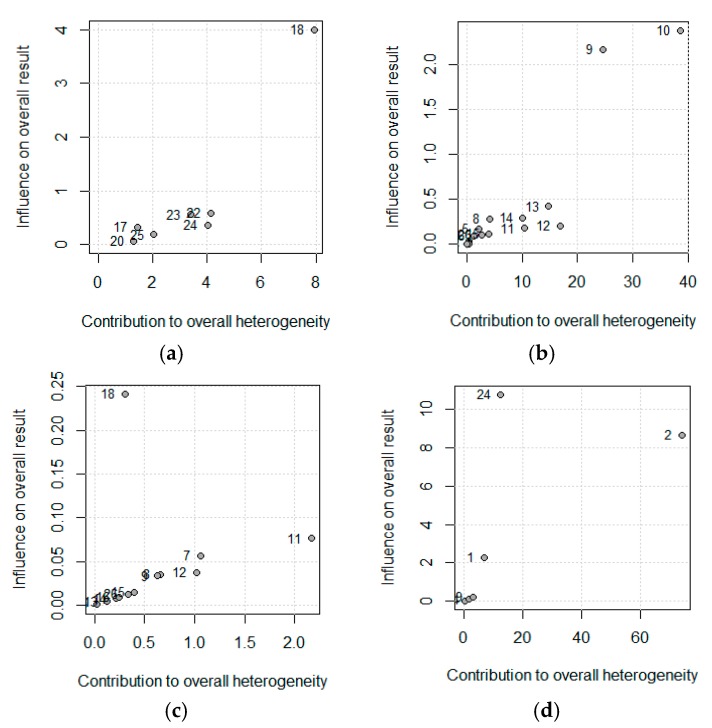
Baujat plot for (**a**) Crohn’s Disease Activity Index (CDAI), (**b**) Albumin (**c**) Body Weight, and (**d**) Postoperative complications. The correspondence between the study and the number is shown in Table 2 (Id, Omitting).

Figure 4 shows this influence through the Baujat plot. The numbers shown in the figure correspond to the articles shown in the table in the ID column. Notice that the studies 18, 10, and 2 correspond to Greenberg et al., Fasth (b) et al., and Elson et al.

**Figure 5:**
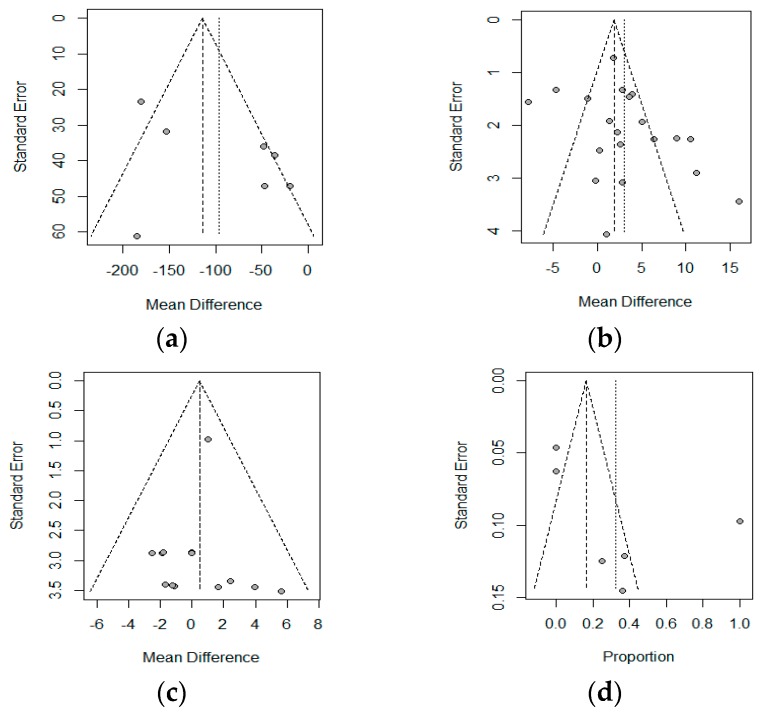
Funnel plot for: (**a**) Crohn’s Disease Activity Index (CDAI), (**b**) Albumin (**c**) Body Weight, and (**d**) Postoperative complications. The correspondence between the study and the number is shown in Table 2 (Id, Omitting).

A Funnel Plot represents the effects observed in the different studies (x-axis), and the standard error (y-axis). In the absence of heterogeneity and publication bias, the dots shown in the funnel plot should jointly adopt the aspect of a funnel, with the wider part corresponding to the smaller and more precise studies. A lack of symmetry could be due to this publication bias. The funnel plot is shown in Figure 5, and a lack of symmetry can be observed. Therefore, the non-parametric analysis proposed by Duval and Tweedie to analyze this asymmetry should show a lack of articles, and therefore, a publication bias. The results of this non-parametric analysis for the fixed-effects model and the random-effects model are shown in Table 4. These results show a possible publication bias in the albumin if a fixed-effects model is assumed; however, the random-effects models do not show this bias. The Copas analysis shows a possible publication bias and suggests that the benefits of PN on albumin could decrease from 3.01 to 2.0.

With respect to the meta-regression, the results are shown in Table 5. There is a dependence of the CDAI score and albumin levels with the observed moment; we have to wait some days for confirming the effects of the PN (*p* < 0.01).

## 4. Discussion

Our systematic review included a total of 15 clinical trials, which compiled information from 557 individuals with IBD, and who had an intervention with PN. All the studies had a broad reach, and within the diverse effects found, BW, albumin, COM, and CDAI were the most common, allowing us to conduct a meta-analysis to arrive at more complete conclusions.

PN implies the intravenous administration of a mix of macronutrients, micronutrients, and electrolytes [47], and its main objective for IBD is to achieve bowel rest, correct nutritional deficits, and the elimination of antigenic stimuli in the mucosa [48]. PN is commonly used during the acute inflammatory phase in patients who are experiencing malnutrition, such as undernourishment [49]. This undernourishment could be a factor that affects micronutrient deficiency [50,51]. The results from the systematic review show that the administration of PN significantly improved the levels of ESR [35,38], cholesterol [46], total phospholipids [46], and serum albumin [37,38,39,40,44,46], without producing clinical symptoms of hypoglycemia, independent of the method of interruption [43]. This improvement of the albumin is mirrored in the results of the meta-analysis. The meta-regression performed showed that the improvement could be greater a few days after the intervention. 

The most common type of under-nourishment in patients with IBD was protein-energetic malnutrition, mainly shown with weight loss [52,53]. This malnutrition could worsen due to diverse surgical interventions that are necessary for emerging situations or when the medical treatment fails [54]. Therefore, the nutritional support should be carefully chosen during the treatment and before the surgery, based on a plan that is customized according to the patient [55]. Some authors declare that PN results in an increase in BMI, helping to correct the individual’s malnutrition [23,56]. We have not collected the BMI; however we have identified the BW, which are equivalent terms in adults, and the meta-analysis did not show the existence of a change in BW in patients with IBD when administering PN. 

The CDAI, developed by Best et al. [57], measures the activity of the disease in patients with CD, with high values indicating a high activity of this pathology. Therefore, a reduction of this index indicates an improvement. The clinical trials conducted showed improvements in this index, but while these were significant in Jones [38], Greenberg et al. [39], Wright et al. [41], the results in Okenga [44] were not. The meta-analysis shows a significant reduction of the values found for the CDAI, and this decrease is accentuated days after the application of PN. These results are in agreement with diverse expert researchers, who declare that PN could provide, along with a possible administration of drugs such as infliximab, an improvement in this pathology [54,58,59].

Despite the accessibility to immunosuppressive drugs, antibiotic treatments, and fecal microbial transplantation, patients experience a high rate of relapse of malabsorption due to intestinal insufficiency [60]. In the case of individuals affected with CD, more than half are subjected to some surgery, such as bowel resection within 10 years after the diagnosis, and a third of them require a resection within the following five years [60]. This is the reason why PN could be fundamental with respect to the survival of the patient, as its management has drastically improved in the last 10 years, and the rate of related complications has notably decreased [60].

Likewise, the role of PN in postoperative complications is controversial. A recent meta-analysis has shown that the pre-surgery nutritional supplementation reduced posterior complications after the surgery in patients with CD, and more specifically, the TPN showed a tendency of being higher than the standard of care without nutritional support, but without statistical significance [61]. Hypoalbuminemia is associated with more postoperative complications, and it is sometimes a contraindication for surgery that requires anastomosis without a protective ileostomy [62]. In our qualitative synthesis with respect to the TPN, the results by Jacobson [46] concluded that it could be recommended for reducing the risk of suffering from postoperative complications until achieving clinical remission, and Yao et al. [45] declare that the perioperative PN may improve humoral immunity, reverse malnutrition and facilitate the rehabilitation of the patient. However, Fasth et al. [36] indicate that the administration of postoperative NPT does not result in a reduction of the complication rate after the surgery, although this difference could be due to the small sample utilized in this study. Our meta-analysis showed that the postoperative complications utilizing PN exist, although the proportion is low.

The term bowel rest has been frequently linked to the use of PN with active IBD or important complications such as the control of sepsis or imminent surgical procedures, and it is also theoretically attractive because of the expectation that it could improve bowel inflammation by alleviating mechanical trauma, bowel secretions, and antigenic challenge of the foods [35,63]. On the contrary, the results by Jones [38] and Dickinson et al. [33] show that there are no differences in patients with CD treated with either EN or TPN, and in patients with IBD treated solely with hydration or TPN. According to Abad-Lacruz et al. [40], and Wright et al. [41], Gonzalez-Huix et al. [42], and Greenberg et al. [39], EN results in significantly less frequent abnormalities in the LFT than TPN in patients with IBD, the PN with bowel rest does not show evidence of having a better impact on the remission than EN in patients with active CD, likewise, the EN is safer, cheaper and nutritionally effective in severe attacks as compared with TPN, and there were no differences in the remission and activity of patients with active CD. 

All of this coincides with diverse studies and clinical practice guides, which indicate that bowel rest is not necessary when nutritional therapy is utilized for managing the patients [48,64,65]. Therefore, they should be allowed to eat “ad libitum” when medical therapy is prescribed and when different nutritional regimes exist through which clinical remission and repair of the mucosa can be achieved [15,48,64,65,66].

Also, it has recently been shown that there is a high load of underfeeding, orders of “nil per os” or a diet with clear liquids, which is unjustified for patients who are hospitalized with CU, especially for patients admitted without evidence of an objective flare of the disease that could be provoking iatrogenic malnutrition, so that bowel rest and the nutritional treatment should be given special attention [67].

Despite being the first systematic review that deals with the general effects of PN on adult patients with IBD, this article is not exempt from limitations. It is possible that the CONSORT questionnaire was not the best for evaluating the NRCCT, and UNRCT reviewed; however, this limitation has been tried to be avoided by adjusting the items of this tool to the type of study, as no questionnaire was found that evaluated the RCCT, the NRCCT and the UNRCT [26,68]. Also, most of the studies were somewhat old, with the most current one being from 2012, which could have reduced the score of this tool on the methodological quality due to the lack of standard criteria at the time the clinical trials were conducted. The UC and CD data have been combined to develop the meta-analysis for the variables BW, albumin, and postoperative complications due to the low number of studies that separated these diseases to elaborate their results. However, these clinical entities have different clinical courses.

The results derived from this work could help in clinical practice, to help the health professionals with the creation of a guide oriented towards evaluating the addition of TPN within the set of medical therapies for an adult patient diagnosed with IBD. However, as future lines of research, the use of TPN with the said patients should be addressed, having in mind their quality of life, the manner of administration, and the composition of the nutritional therapy in all the surgical procedures possible.

## 5. Conclusions

PN has shown to have efficacy for the treatment of IBD and is compatible with other medicines. The CDAI and albumin improve, although the effect of PN is greater after a while. However, the effect on the albumin could be less than the observed value in the meta-analysis, due to possible publication bias. The body weight does not change after intervention. Postoperative complications utilizing PN has been observed, although the proportion is low. More studies specifically referred to UC and CD are needed to develop more concrete clinical results.

## Figures and Tables

**Table 1:** Main results of the systematic review.

Author	Study	n/Age	Disease	P/d	CC	Treatment	Variables	Main Results
Goode et al., 1976 [32]	UNRCT	8/NI M NI F NI	SUR CD	360	GB	Intravenous feeding through an elemental diet in the whole sample	BW, nitrogen balance, TBF measured by anthropometry, TBK.	3 (37.5%) preoperative patients were able to restore 10% of their lost lean tissue per month with an ED, which contained 12 g/day of nitrogen. In the five (62.5%) postoperative patients who had a successful resection, the rate of restoration of the lean tissue mass was 18.5% per month with a nitrogen intake in the elemental form of 10 g/day. In all the patients given an ED, it was possible to restore all the lean tissue loss.
Dickinson et al., 1980 [33]	RCCT	40/41,75 M 13 F 27	ACT IBD EG/CG 20/20	18	GB	EG: 2–3 L/day of “Freamine^®^” intravenous solution (dextrose, electrolytes, and vitamins) via the central venous catheter CG: blood, albumin, and, normal diet plus vitamins (Multibionta, Merck). All groups: Prednisone 40 mg/day	BW, TBN, patients to colectomy, patients who reduce prednisone to 10 mg/day	The mean time to reduce prednisone to 10 mg/day was 23.7 ± 6.0 days for the control patients and 21.2 ± 3.7 days for the EG, respectively. Six (30%) patients in the CG and nine (45%) patients in the EG came to surgery. In CG, 11 (55%) patients responded medically and in EG 10 (50%) patients. The CG lost significantly 108 g of TBN (7.3% of body protein mass) while in the EG, there was no change. However, the weight gain observed in both groups did not reflect changes in TBP.
Elson et al., 1980 [34]	UNRCT	30/23 M 14 F 16	ACT IBD CD: 20 UC: 10	CD:36 UC:21	US	TPN: Synthetic formulation (Freamine^®^) and a protein hydrolysate (Amigen^®^). Ampule multivitamins, folic acid, and water.	Weight gain, Nitrogen balance, albumin and symptoms such as abdominal pain, diarrhea, fever, rectal bleeding, fistula, obstruction, abdominal mass, proctoscopy change, hematocrit, and blood transfusion requirement.	CD: All patients gained weight. Weight changed from 6.6 kg of loss to 6.8 kg of gain. There was a small increase in albumin from 2.7 to 3.2. 13/20 (65%) patients had a positive clinical response. TPN improved symptoms such as diarrhea and pain and a sense of well-being, but not the rest. Nonresponse was found in 3/4 (75%) patients receiving TPN primarily to heal fistulas. Although complications were frequent and 10 (50%) patients were eventually operated. UC: 9/10 (90%) patients gained weight. Weight changed from 9.3 kg of loss to 3.6 kg of gain after TPN. 4/10 (40%) patients had a positive response to TPN. Complications were common, and 6/7 patients were having a colectomy.
McIntyre et al., 1986 [35]	RCCT	47/36 M 19 F 28	ACT IBD EG/CG 27/20	7	GB FR	EG: PN and water. CG: oral diet. All groups: 60 mg/day prednisolone.	The operation and mortality rates, clinical, and laboratory data, such as stool weight, albumin, HB, ESR, WBC, etc.	There was no significant difference between the groups for any parameter measured. Within each group the stool weight and stool frequency decreased significantly in EG (*p* < 0.01), between days 0 and 7 but not in CG. The ESR was significantly reduced in both groups (*p* < 0.01). In CG, serum albumin concentrations increased significantly compared with day 0 (*p* < 0.02). 11 (40.7%) patients in EG and 5 (25%) patients in CG required surgery. There were two deaths during the hospital admission, one in each group. Surgical treatment was required by 14 of 27 patients with UC but none of 16 with CD (*p* < 0.01).
Fasth et al., 1987 (a) [36]	RCCT	92/51 M NI F NI	SUR Cancer/IBD(REM)/IBD (ACT) 50/16/26 EG/CG 48/44	9.7	SE	EG: TPN composed of amino acid solution, 20% fat, and 10% dextrose. CG: a 10% dextrose and electrolytes solution	Postoperative complications	The total mortality was 2 (2.2%) in 92 operations. Forty-eight early complications after major colorectal surgery were diagnosed in 33 (36%) patients in the whole sample. There was no morbidity associated with the central venous lines or the TPN treatment per se. The overall complication rate in both groups was similar, without significant differences.
Fasth et al., 1987 (b) [37]	RCCT	92/51 M NI F NI	SUR Cancer/IBD(REM)/IBD (ACT) 50/16/26 EG/CG 48/44	9.7	SE	EG: TPN composed of amino acid, 20% fat, and 10% dextrose. CG: a 10% dextrose and electrolytes solution	Albumin, BW, TBK, TSF, TBW, nitrogen balance	The cumulative nitrogen balance after one week was +0.1 g in TPN-patients, and −47.3 g in controls (*p* < 0.001). The difference in relative weight loss between TPN and control patients was statistically significant at 1, 2, 4, 8, and 24 weeks (group cancer), at 1, 2, and l 4 weeks (group IBD-REM) and at 2 weeks (group IBD-ACT). The reduction in TBK was significantly less in the TPN-patients of group cancer and IBD-REM than in the controls. In all three groups, TPN-patients had a higher mean value than controls at every postoperative measurement for TSF. This difference between TPN and controls reached statistical significance only in group IBD-REM at 1 and 4 weeks, and in group IBD-ACT at 2 weeks. After the first week, the albumin in groups cancer and IBD-REM increased towards the preoperative levels. The increase was faster in TPN patients, and a statistically significant difference was found in group IBD-REM at 8 and 24 weeks.
Jones 1987 [38]	RCCT	36/31 M 9 F 27	ACT CD EG/CG 19/17	14	GB	EG: TPN with 0.17–0.3 g N × kg^−1^, electrolytes, all vitamins, and water. CG: 300–500 g of ED “EO28”	CDAI, albumin, orosomucoid, ESR.	No difference was detected in the success rate, the speed of achieving remission, the changes in CDAI, erythrocyte sedimentation rate (ESR) and serum albumin between the two groups. The fall in the CDAI in both groups was significant (*p* < 0.01).
Greenberg GR et al., 1988 [39]	RCCT	51/30 M 25 F 26	ACT CD TPNG/ENG/PPNG 17/19/15	21	CA	TPNG: TPN more water, plus an ampule of vitamins per day. ENG: formula diet “Precision-Isotonic”. PPNG: Unrestricted diet and a partial protein/calorie PN.	CDAI, nutritional assessment and biochemical measurements (hematocrit, blood glucose, electrolytes, creatinine, magnesium, and albumin).	The average CDAI decreased (*p* < 0.01) with no significant differences between groups. Remission rates to discharge were similar among the three groups: 12 (70.6%) patients in TPNG, 11 (57.9%) patients in ENG, and 9 (60%) patients in PPNG (X^2^ 1.42 and 1.15; *p* = n/s). Remission rates of 42% in TPNG, 55% in EN and 56% in PPNG at 12 months were equivalent and not influenced by the type of nutritional support initially administered. In the whole sample, at 12 months, 18 (35%) patients required surgery, 17 (34%) were medically treated for relapse, and 16 (31%) had sustained remission.
Abad-Lacruz A et al., 1990 [40]	RCCT	29/32 M 15 F 14	ACT IBD PG/TPNG 16/13	17.4	ES	PG: Polymeric diet high in nitrogen “UNIASA” by nasogastric tube. TPNG: Specific total PN by a central vein.	Biochemical measurements (serum albumin, GGT, ALT, AST, etc.) and VHAI and the Truelove and Witts index were measured.	PG had a significant increase in albumin concentration (32 ± 1 to 38.2 ± 1.6 g/liter; *p* < 0.01). There was lower disease activity in both groups (3.31 ± 0.15 to 2.31 ± 0.24, *p* < 0.05 in PG; and 3.38 ± 0.21 to 2.61 ± 0.27, *p* < 0.05 in TPNG). 8 (5 CD and 3 UC) of 13 patients (61.5%) in the TPNG group developed abnormalities in LFT, while in the PG group, it only occurred in 1/16 (6.2%) patients (*p* = 0.002).
Wright RA et al., 1990 [41]	RCCT	11/NI M 7 F 4	ACT CD TENG/ TPNG 6/5	14	US	TENG: Total elemental enteral feeding “Vital” TPNG: Total peripheral PN.	CDAI, standard anthropometric parameters, nitrogen balance studies and chemical profiles.	CDAI improved significantly in both groups. Plasma transferrin levels and TLC improved in the TENG group (*p* < 0.05). No significant differences in weight gain. TLC improved in all patients receiving EN but did not change significantly in those receiving PN. Improvement in serum transferrin levels correlated positively (*p* < 0.05) in patients receiving EN but not in PN.
González-Huix et al., 1993 [42]	RCCT	42/33,25 M 21 F 21	ACT UC TENG/TPNG 22/20	16	ES	TENG: Polymeric EN, administered intragastrically. TPNG: All in one admixture PN with a composition similar to that of TEN.	TSF, MAMC, BW, %IBW, albumin, complications attributable to ANS, score Truelove, and Witts.	No significant changes were observed in anthropometric parameters at the end of either TENG or TPNG treatment. However, a significant increase in albumin concentration was observed in the TENG (*p* = 0.015). As a consequence, the median increase in albumin values was significantly higher in patients on TENG 16.7% (−0.5 to +30.4%) than on TPNG 4.6% (−12.0 to+ 13.7%) (*p* = 0.019). Ten patients in each group required colectomy. Postoperative infections occurred significantly more often in patients on TPNG than in those on TEN (*p* = 0.028). There were significantly more ANS-related complications in the TPNG than in the TENG (35% vs. 9%; *p* = 0.046).
Eisenberg et al., 1995 [43]	RCCT	12/37,7 M 6 F 6	ACT IBD/SBF/II 10/1/1	1.5	US	AS: Abrupt interruption TPN with steroids. AWS: Abrupt interruption TPN without steroids. TS: Taper interruption TPN with steroids. TWS: Taper interruption TPN without steroids.	Glycemic symptom by questionnaire, pulse, blood samples for glucose, insulin, growth hormone, cortisol, epinephrine, norepinephrine, and glucagon.	Plasma concentrations of glucose decreased significantly (*p* < 0.001) after tapered and abrupt discontinuation of TPN infusion in all 12 patients without differences between these methods, and no patient experienced clinical symptoms of hypoglycemia. Mean norepinephrine and epinephrine levels were only slightly higher after abrupt discontinuation, compared with tapering of TPN, without significant differences. Physiologic responses were also not statistically different after the two methods of discontinuation. Mean levels of insulin decreased significantly after discontinuation of TPN (*p* < 0.001), but peripheral glucagon levels remained essentially unchanged in all groups. No statistical difference between methods for cortisol and growth hormone.
Ockenga et al., 2005 [44]	RCCT	24/35 M 15 F 9	ACT IBD TPN+/TPN- 12/12	21.5	DE	TPN+: TPN with alanyl-glutamine “Dipeptamin^®^”. TPN-: TPN with a standard aminoacid solution “Aminoplasmal^®^”. All patients: mesalazine and prednisolone 0.5–1 mg/kg/day or azathioprine.	LOS, CDAI, BMI, blood sample for HB, hematocrit, WBC, albumin, CRP, urea, and AA. Intestinal permeability,	Glutamine plasma levels did not change significantly in either group throughout the study. BMI, albumin level, citrulline, or arginine levels did not change significantly in either group. Glutamine supplementation did not appear to produce any significant difference in D-lactulose/xylose ratio (TPN+: 0.01 vs. TPN-: 0.02; *p* = 0.82) and it exerted no specific effect on CDAI, WBC, or total lymphocyte count compared to standard TPN. The duration of TPN and LOS did not differ significantly between groups.
Yao et al., 2005 [45]	NRCCT	32/29 M 19 F 13	SUR CD EG/CG 16/16	21	CN	EG: Perioperative PN CG: intravenous transfusions containing energy 20 kcal/kg/day, normal water, and diet	Serum IgM, IgG, and IgA, LF, bilirubin levels, BMI, BW, BH, postoperative complications.	IgM levels decreased significantly three weeks after surgery only in EG. BMI increased significantly in EG, and no change in CG. There were no significant changes in concentrations of IgG and IgA. The overall complication rates of both groups were similar.
Jacobson 2012 [46]	NRCCT	120/35 MEG 4 FEG 11	SUR CD EG/CG 15/105	46	SE	EG: TPN preoperative (amino acids “Vamin^®^”, carbohydrates, fat emulsion “Intralipid ^®^”, electrolytes, trace elements “Addamel^®^”, and vitamins “Soluvit^®^ and Vitalipid^®^”). CG: Patients operated without preoperative TPN.	Early postoperative complications and biochemical blood parameters.	During the preoperative TPN, all the cases in EG displayed clinical remission of CD. There was no significant postoperative complication in the EG, whereas there were 29 (27.6%) patients with postoperative complications in CG. This is a higher rate of complications (*p* < 0.05) than in EG. There was a significant increase in the variables with TPN preoperative: BW, BMI, albumin, haptoglobin, cholesterol, triiodothyronine, Ig A, Ig M, phospholipids total, lecithin. There was also a significant decrease in the variables: White cell count, haptoglobin, and triglycerides.

%IBW: Percentage of ideal body weight. AA: Plasma amino acid concentration. ACT: Active disease. ALT: Alanine aminotransferase. ANS: Artificial nutritional support. AST: Aspartate aminotransferase. BMI: Body mass index. BW: Bodyweight. CC: ISO Country Codes. CD: Crohn Disease. CDAI: Crohn’s Disease Activity Index. CRP: C-reactive protein. ED: Elemental diet. EG/CG: Experimental and Control Group. EN: Enteral Nutrition. ESR: erythrocyte sedimentation rate. F: Female. GGT: γ-glutamyltransferase. HB: Hemoglobin. HEEH: Home elemental enteral hyperalimentation. IBD: Inflammatory Bowel Disease. IBD: Inflammatory Bowel Disease. IBW: Ideal body weight. II: Intestinal inertia. LFT: Liver function test. LOS: Length of hospital stay. M: Male. MAMC: Mid-arm muscle circumference. N: Nitrogen. NI: Not indicated. NRCCT: Non-randomized controlled clinical trials. P/d: Period (days). PN: Parenteral nutrition. RCCT: Randomized controlled clinical trials. REM: Disease in remission. SBF: Small bowel fistula. SUR: Surgery. TBF: Total body fat. TBK: Total body potassium. TBN: Total body nitrogen. TBP: Total body protein. TBW: Total body water. TEN: Total enteral nutrition. TLC: Total lymphocyte count. TPN: Total parenteral nutrition. TSF: Triceps skinfold thickness. UC: ulcerative colitis. UNRCT: Uncontrolled and non-randomized clinical trial. VHAI: Van Hees Activity Index. WBC: White blood cells.

**Table 2:** Methodological quality analysis according to the CONSORT guide [25] for reporting clinical trials.

Studies	1	2	3	4	5	6	7	8	9	10	11	12	13	14	15	16	17	18	19	20	21	22	23	24	25	Total Score	(%)
Goode et al., 1976 [32]	0	1	0	0.5	0	0.5	0	NA	NA	NA	NA	0.5	NA	0	0	0	0	NA	0	1	1	1	NA	NA	NA	5.5/16	34.4
Dickinson et al., 1980 [33]	0	1	0.5	1	1	0.5	0	0	0	0	0	0	1	1	1	1	0	0	1	1	1	1	0	0	0	12/25	48
Elson et al., 1980 [34]	0	1	0.5	1	1	0.5	0	NA	NA	NA	NA	0	NA	0.5	1	1	0	NA	1	1	1	1	NA	NA	NA	10.5/16	65.6
McIntyre et al., 1986 [35]	0	1	0	0.5	1	1	0	1	0	0	0	0.5	1	1	1	1	0	1	1	1	1	1	0	0	0	14/25	56
Fasth (a) et al., 1987 [36]	0	1	0.5	0.5	1	1	0.5	0	0	0	0	0.5	1	1	0	1	0.25	1	1	1	1	1	0	0	1	14.25/25	57
Fasth (b) et al., 1987 [37]	0	1	0.5	0.5	1	0.5	0	1	0	0	0	0.5	1	1	1	1	0.25	0	1	1	1	1	0	0	1	14.25/25	57
Jones 1987 [38]	0.5	1	0.5	1	1	0.25	0	0	0	0	0	0	1	1	1	1	0.25	0	1	0	1	1	0	0	1	12.5/25	50
G.R. Greenberg et al., 1988 [39]	0	1	0.5	1	1	1	0	1	1	0	1	1	0.5	1	1	1	0.25	0	1	1	1	1	0	0	0	16.25/25	65
A. Abad-Lacruz et al., 1990 [40]	0.5	1	0.5	0.5	1	0.5	0	1	0	0	0	1	1	0.25	1	1	0.5	0	1	0	1	1	0	0	0	12.75/25	51
R. A. Wright et al., 1990 [41]	0	1	0.5	0.5	1	0.5	0	0.5	0	0	0	1	0.5	0.75	0	1	0.25	0	0	1	1	1	0	0	1	11.5/25	46
González-Huix et al., 1993 [42]	0	1	0.25	0.5	1	0.5	0	0	0	0	0	1	1	1	1	1	0.25	0	1	0	1	1	0	0	0	11.5/25	46
Eisenberg et al., 1995 [43]	0	1	0.5	1	1	0.5	0	0	0	0	0	1	1	0	0	1	0.5	0	1	0	1	1	0	0	1	11.5/25	46
Ockenga et al., 2005 [44]	0.5	1	0.5	1	1	0.5	1	1	1	0	1	1	1	0.5	1	1	0.25	0	1	1	1	1	0	0	1	18.25/25	73
Yao et al., 2005 [45]	0.5	1	0	0.5	1	0.5	0	NA	NA	NA	NA	1	1	0	1	1	0.25	NA	1	0	1	1	NA	NA	NA	10.75/17	63.2
Jacobson 2012 [46]	0.5	1	0	0.5	1	0.5	0	NA	NA	NA	NA	1	1	0.5	1	1	0.25	NA	1	1	1	1	NA	NA	NA	12.25/17	72.1

NA: Not applicable.

**Table 3:** Influence analysis in a meta-analysis using the leave-one-out method (Random effect).

			Meta-Analysis for: Effect Size (%Heterogeneity)			
ID	Omitting	n	CDAI	Albumin	BW	COM
1	1980 Elson et al. [34]	10				39.6% (95.5%)
2	1980 Elson et al. [34]	6				16.0% (75.0%)
3	1987 Fasth (a) et al. [36]	12				34.2% (95.9%)
4	1987 Fasth (a) et al. [36]	11				32.0% (95.9%)
5	1987 Fasth (b) et al. [37]	12		3.0 (86.1%)	0.49 (0.0%)	
6	1987 Fasth (b) et al. [37]	12		3.0 (86.2%)	0.49 (0.0%)	
7	1987 Fasth (b) et al. [37]	12		3.1 (86.3%)	0.62 (0.0%)	
8	1987 Fasth (b) et al. [37]	12		3.3 (85.8%)	0.59 (0.0%)	
9	1987 Fasth (b) et al. [37]	12		3.5 (82.8%)	0.59 (0.0%)	
10	1987 Fasth (b) et al. [37]	12		3.6 (80.1%)	0.49 (0.0%)	
11	1987 Fasth (b) et al. [37]	11		2.6 (85.1%)	0.28 (0.0%)	
12	1987 Fasth (b) et al. [37]	11		2.5 (84.3%)	0.34 (0.0%)	
13	1987 Fasth (b) et al. [37]	11		2.6 (84.5%)	0.42 (0.0%)	
14	1987 Fasth (b) et al. [37]	11		2.7 (85.1%)	0.52 (0.0%)	
15	1987 Fasth (b) et al. [37]	11		2.8 (85.9%)	0.55 (0.0%)	
16	1987 Fasth (b) et al. [37]	11		3.1 (86.3%)	0.53 (0.0%)	
17	1987 Jones et al. [38]	16	−85.8 (77.8%)	3.2 (86.3%)		
18	1988 Greenberg et al. [39]	19	−78.6 (59.4%)	3.1 (86.3%)	0.04 (0.0%)	
19	1990 Abad-lacruz et al. [40]	13		3.2 (86.3%)		
20	1990 Wright et al. [41]	5	−86.2 (78.1%)			
21	2005 Yao et al. [45]	16				31.7% (94.7%)
22	2005 Ockenga et al. [44]	9	−107.2 (74.4%)	3.1 (86.3%)		
23	2005 Ockenga et al. [44]	9	−105.3 (75.4%)	3.0 (86.3%)		
24	2005 Ockenga et al. [44]	10	−108.3 (74.8%)	3.2 (86.3%)		
25	2005 Ockenga et al. [44]	10	−104.0 (77.3%)	3.0 (86.3%)		
26	2012 Jacobson [46]	14		2.9 (86.0%)	0.39 (0.0%)	39.7% (94.7%)
	Pooled estimate		−96.7 (75.3%)	3.0 (85.6%)	0.46 (0.0%)	32.6% (94.9%)

CDAI: Crohn´s Disease Activity Index; BW: Body Weight, COM: Postoperative complications.

**Table 4:** Number of studies that should be added and the estimated effect size.

	Trim-and-Fill Method				Copas Method	
	Fix Model		Random Model		Random Model	
	Ns	Effect Size Estimated 95%CI	Ns	Effect Size Estimated 95%CI	Ns	Effect Size Estimated 95%CI
CDAI	2	−128.5 [−184.7; −72.3]	0	Unchanged	0	Unchanged
ALB	6	0.58 [−1.57; 2.74]	0	Unchanged	5	2.0 [0.16; 3.84]
BW	2	0.76 [−0.45; 1.98]	2	0.76 [−0.45; 1.98]	0	Unchanged
COM	3	0.04 [0.00; 0.37]	0	Unchanged	0	Unchanged

Ns: Number of studies added, CDAI: Crohn’s Disease Activity Index, ALB: Albumin, BW: Body Weight, COM: Postoperative complications.

**Table 5:** Meta-regression.

Result		Co-Variable	Test of Moderators	
	**Intercep**	Surgery *	**QM**	***p*-Value**
**CDAI**	−96.68	---	---	---
**ALB**	1.39	2.47	1.02	0.312
**BW**	1.00	−0.96	0.54	0.461
	**Intercep**	**Treatment (days)**	**QM**	***p*-Value**
**CDAI**	−357.50	13.43	2.30	0.129
**ALB**	2.60	0.03	0.06	0.799
**BW**	−0.84	0.08	1.028	0.310
**COM**	0.63	−0.01	1.56	0.212
	**Intercep**	**Observed Moment (days)**	**QM**	***p*-Value**
**CDAI**	47.07	−10.77	7.95	0.005
**ALB**	0.54	0.05	7.42	0.006
**BW**	−0.16	0.01	1.04	0.307

* Basis Group, No Surgery. CDAI: Crohn’s Disease Activity Index, ALB: Albumin, BW: Body Weight, COM: Post-operative complications.
